# Wave Measurements Using GPS Velocity Signals

**DOI:** 10.3390/s110101043

**Published:** 2011-01-18

**Authors:** Dong-Jiing Doong, Beng-Chun Lee, Chia Chuen Kao

**Affiliations:** 1 Department of Marine Environmental Informatics, National Taiwan Ocean University, 2, Beining Rd., Keelung 20224, Taiwan; 2 Department of Environmental and Hazards-Resistant Design, Huafan University, 1, Huafan Rd., Shihding Dist., New Taipei City 22301, Taiwan; 3 Department of Hydraulic and Ocean Engineering, National Cheng Kung University, 1, Da-Hsueh Rd., Tainan 70101, Taiwan; E-Mail: kaoshih@narl.org.tw

**Keywords:** GPS, wave measurement, directional wave spectrum

## Abstract

This study presents the idea of using GPS-output velocity signals to obtain wave measurement data. The application of the transformation from a velocity spectrum to a displacement spectrum in conjunction with the directional wave spectral theory are the core concepts in this study. Laboratory experiments were conducted to verify the accuracy of the inversed displacement of the surface of the sea. A GPS device was installed on a moored accelerometer buoy to verify the GPS-derived wave parameters. It was determined that loss or drifting of the GPS signal, as well as energy spikes occurring in the low frequency band led to erroneous measurements. Through the application of moving average skill and a process of frequency cut-off to the GPS output velocity, correlations between GPS-derived, and accelerometer buoy-measured significant wave heights and periods were both improved to 0.95. The GPS-derived one-dimensional and directional wave spectra were in agreement with the measurements. Despite the direction verification showing a 10° bias, this exercise still provided useful information with sufficient accuracy for a number of specific purposes. The results presented in this study indicate that using GPS output velocity is a reasonable alternative for the measurement of ocean waves.

## 1. Introduction

Field measurement of ocean waves is necessary for the calibration and validation of wave models in modern coastal technology, as well as for the collection of information for engineering construction projects, port operations, and disaster prevention. Ultrasonic, hydraulic and accelerometer devices for measuring waves are already in practical use. In ultrasonic and hydraulic devices, waves are measured by measuring of distance to the surface of the sea through the emission of ultrasonic waves from an observation device anchored at the sea bottom. A more common and robust way of measuring waves that does not require a fixed platform is using a buoy to record the motion of the water surface. The motion of the buoy provides a time history of the water elevation for that location, which can be used to calculate significant wave heights and periods. Modern data buoys usually measure their movement in three dimensions in providing simultaneous information concerning wave direction.

**Figure 1.**
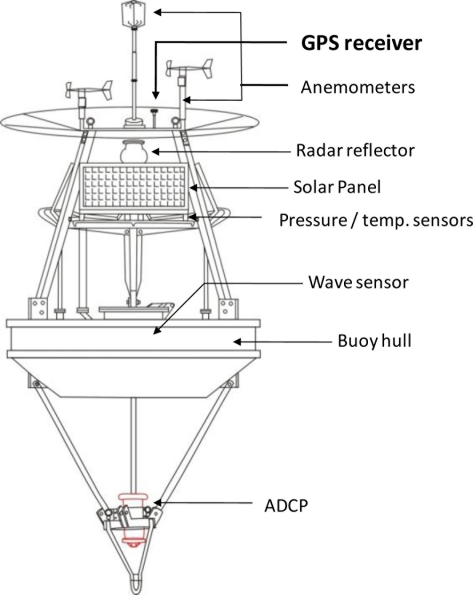
Sketch of the GPS buoy.

Fifteen data buoys are deployed and operated along the Taiwanese coastline [1]. The buoys are 2.5 meters in diameter. A GPS (Global Positioning System) device is mounted on each buoy to monitor their position, as shown in Figure 1. These data buoys are typically positioned off of areas that are prone to coastal flooding or along routes to recreational islands. Over the last 10 years, an average of seven typhoons have approached Taiwan’s waters each year. Wave information is necessary for numerical modeling applied in the coastal warning system. Applying buoy-mounted GPS devices to measure waves is an alternative to the accelerometer, and discovering low-cost devices for the operation of coastal ocean monitoring networks is always a direction to work towards.

Following the recent development of GPS technology, many studies on wave measurement through the use of GPS have been carried out by researchers such as Kato *et al.* [2], Jeans *et al.* [3], Fujita *et al.* [4], Yoo *et al.* [5], Nagai *et al.* [6], Harigae *et al.* [7] and Hou *et al.* [8]. Due to its highly accurate localization many studies have used the RTK (Real Time Kinematics) method as a positioning system for the GPS observation equipment, but it requires a fixed reference point and, therefore, is restricted regarding the distance between the GPS buoy and base station. The differential-GPS (DGPS) is nowadays also used for wave measurement. However it requires an additional GPS reference station on shore, restricting it to only near-shore applications. De Vries *et al.* [9] presented a new Datawell GPS wave buoy using a single GPS receiver. They derived the motion of the GPS by integration of moving speed of the buoy that computed from Doppler-shifted frequency. Rossouw *et al.* [10] studied on the data quality control for a directional wave buoy using differential GPS technology. They showed the influence of missing data influenced the results from GPS measurement. Isshiki *et al.* [11] developed point a precise variance detection (PVD) method to extract changes in an observation point and, therefore, eliminated the need for a base station. Hou *et al.* presented a velocity integration method to obtain precise velocity information of GPS buoys, which was applied to estimate the position of the buoys [8]. This process requires a satellite velocity and can therefore only be used for post-analysis. Recently, Bender *et al.* [12] evaluated the performance of the determination of wave heights and periods derived from a dual frequency buoy-mounted GPS. They concluded that when the motion of the GPS antenna is properly understood as the motion of the buoy deck and not the true vertical motion of the sea surface, the GPS wave heights are as reliable as a strapped-down 1D accelerometer. They also used the post-processed data technique.

Real-time computational capability is necessary for an operational ocean measurement system. In this paper, we have attempted to extract GPS velocity data to estimate wave parameters, as a transformational relationship exists between the velocity spectrum of water particles in water and the displacement spectrum [13]. We have applied this idea to derive wave parameters from fluctuations in a GPS buoy, and carried out laboratory experiments and field tests to verify the concept.

## 2. Methodology

### 2.1. Derivation of Displacement Spectrum

Most modern GPS devices output location and elevation data, and current GPS technology has improved the resolution of GPS (receiver) location. However, a number of GPS devices can output velocity as well. This paper applies data regarding GPS output velocity to study derivations in the parameters of ocean waves. GPS receivers obtain two velocity readings for the object. One reading is mean velocity; the other is instant velocity. Mean velocity refers to the time differentiation of object displacement. It is used for objects that do not express large velocity gradients, such as cars or airplanes. Instant velocity and direction are estimated from the Doppler Effect relative to the GPS receiver and the motion of the satellite. A shift in frequency (*f_d_*) is estimated when the frequency of electromagnetic waves emitted and received by the satellite are known, as shown in Equation (1):

(1) $$f_{d}=f_{r}-f_{s}=\frac{-\bar{v_{\mathrm{rs}}}}{c}f_{s}$$

where *f_r_* and *f_s_* are emitted and received frequencies, respectively; ν̄*_rs_* = ν̄*_r_* – ν̄*_s_* is the relative mean velocity of the satellite and receiver; ν̄*_r_* is the mean velocity of the satellite and ν̄*_s_* is the mean velocity of the receiver; and *c* is a constant. The instant velocity of the GPS receiver is obtained from Equation (1) when the Doppler frequency shift and the speed of the satellite movement are known. When a GPS receiver is installed on an orbital motion buoy, the instant velocity is assumed to be the water-particle velocity. However, the satellite velocity is difficult to determine in real time, and must be post-processed, therefore real time measurement of ocean waves cannot be achieved. In this study, data processes in the spectral domain were applied.

The velocity spectrum was derived by a Fourier transform of autocorrelation function of GPS output velocity. The autocorrelation function is defined as:

(2) $$R_{\mathrm{ii}}(\tau )=\underset{T\to \infty }{\text{lim}}\frac{1}{T}\int_{-T/2}^{T/2}V_{i}(t)V_{i}(t+\tau )\mathrm{dt}$$

where *V_i_*(*t*) is the time series of GPS output velocity. *i* = 1, 2, 3 represent vertical, horizontal E-W and horizontal S-N directions, respectively. Applying the Fourier transform to Equation (2), we obtain:

(3) $$S_{v}(f)=\int_{-\infty }^{\infty }R_{\mathrm{ii}}(t)\;\text{exp}\;(-i2\pi \;\mathrm{ft})\;\mathrm{dt}$$

where *S_v_*(*f*) is the velocity spectrum, representing the energy distribution of certain frequency bands in the velocity domain. Because the motion of ocean waves is within a specific frequency band, a band pass filter was applied to obtain the velocity spectrum for wave motion. The filter is shown below:

(4) $$S_{v}^{'}\;(f)=S_{v}\;(f)\cdot H(f)$$

where *S_v_*′(*f*) is the velocity spectrum within the frequency band of the motion of ocean waves. *H*(*f*) is a band pass filter function, which is:

(5) $$H\;(f)=\{\begin{array}{ll} 0 & f>B,\;f<A\\ 1 & A\leq f\leq B \end{array}$$

In this case, *A* = 0.03 and *B* = 0.4 are used in order to satisfy the general properties of ocean waves. The displacement spectrum *S*(*f*) was then derived from velocity spectrum by a transformation function [13] as shown below:

(6) $$S\;(f)=S_{V}^{'}\;(f)\times {\left(2\pi f\right)}^{-2}$$

The significant wave height *H_s_* and the mean wave period *T̄* can then be derived from the displacement spectrum:

(7) $$H_{s}=c\sqrt{m_{0}}$$

(8) $$\bar{T}=\sqrt{m_{0}/m_{2}}$$

where *m*_0_ and *m*_2_ are the moments of zero- and the second orders of the energy distribution. *c* is a constant.

### 2.2. Derivation of Directional Spectrum

Wave energy measured at a point has angular distribution as well as distribution over a range of frequencies. The angular distribution of wave energy is termed directional spreading. Spectral representations including both frequency distribution and the angular spreading of wave energy are known as directional spectra *S(f,* *θ)* (Massel, 1996) [14]. Knowledge of directional spectra is important for coastal engineers. More accurate wave prediction methods can usually be obtained when directional spectra are considered. The directional wave spectrum is usually obtained through a best-fit approach from a set of integral equations involving the directional spreading function [15].

The cross spectrum *ϕ_ij_*(*f*) s the forward Fourier transform of the cross correlation function *R_ij_*(*π*). The cross spectrum is in general complex. The cross correlation function of a waveform function is shown below:

(9) Rij (τ)=limT→∞1T∫−T2T2 ηi (t)ηj (t+τ) dt

where *η*(*t*) is a waveform function, *i*, *j* = 1, 2, 3 … are the measured parameters, such as acceleration, velocity or displacement. In this study, they represent the GPS output velocities in three directions, and *τ* is the lag time. The cross spectrum *ϕ_ij_*(*f*) is therefore derived by Equation (10):

(10) $$\phi_{\mathrm{ij}}\;(f)=\int_{-\infty }^{\infty }R_{\mathrm{ij}}\;(\tau )e^{-2\pi \mathrm{if}\tau }d\tau$$

The complex form cross spectrum can be written as:

(11) $$\phi_{\mathrm{ij}}\;(f)=C_{\mathrm{ij}}\;(f)-{\mathrm{iQ}}_{\mathrm{ij}}\;(f)$$

where the real part of the cross spectrum *C_ij_*(*f*) is called the co-spectrum, and the imaginary part *Q_ij_*(*f*) is the quadrature-spectrum. Longuet-Higgins *et al.* (1963) [16] presented the correlation between cross spectrum and directional spectrum is shown below:

(12) $$\phi_{\mathrm{ij}}\;(f)=\int e^{-{\mathrm{ikx}}_{\mathrm{ij}}}\cdot S\;(f,\;\theta )\;d\theta$$

Isobe *et al.* [17] modified Equation (12) to a general form, shown in Equation (13):

(13) $$\begin{array}{lll} \phi_{\mathrm{ij}}\;(f) & = & \int_{-\pi }^{\pi }H_{i}\;(f,\theta )\cdot \bar{H_{j}}\;(f,\theta )\\ & & \cdot \left\{\mathrm{Cos}\;\left[k\cdot \;\left(x_{\mathrm{ij}}\;\mathrm{Cos}\theta +y_{\mathrm{ij}}\;\mathrm{Sin}\;\theta \right)\;\right]-i\;\mathrm{Sin}\left[k\left(x_{\mathrm{ij}}\;\mathrm{Cos}\;\theta +y_{\mathrm{ij}}\;\mathrm{Sin}\;\theta \right)\right]\right\}\\ & & \cdot S\;(f,\theta )\;d\theta \end{array}$$

where *x_ij_* and *y_ij_* are the distances between measurement sensors; *H_i_(f, θ)* is the transfer function; and *H̄_i_*(*f, θ*) is its conjugate function. The transfer function can be derived by impulse response method or frequency response method. It represents the properties on surface fluctuation correlated to the measured parameters.

The GPS receiver was mounted on a wave following buoy. The output velocities in the heave, surge and sway directions were assumed to be from the same location. Therefore *x_ij_* = *0* and *y_ij_* = *0* were applied to Equation (13) to simply the computation and re-formulated as Equation (14).

(14) $$\phi_{\mathrm{ij}}\;(f)=\int_{-\pi }^{\pi }H_{i}\;(f,\theta ){\bar{H}}_{j}\;(f,\theta )\;S\;(f,\theta )\;d\theta$$

The transfer function and its conjugate function are listed by Isobe *et al.* [17]. The directional spectrum *S*(*f*, *θ*) in Equation (14) is expressed as a finite Fourier series, as shown in Equation (15):

(15) $$S\;(f,\theta )=a_{0}\;(f)+\sum a_{n}\;(f)\;\text{cos}\;n\theta +b_{n}\;(f)\;\text{sin}\;n\theta$$

Because the GPS outputs three-dimensional velocities, six-pair cross-spectra were obtained from Equations (9) and (10). They were used to solve Equations (11), (12) and (15) up to the second order. When the directional spectrum was derived, the wave parameters were obtained by Equations (7) and (8).

## 3. Laboratory Experiments

**Figure 2.**
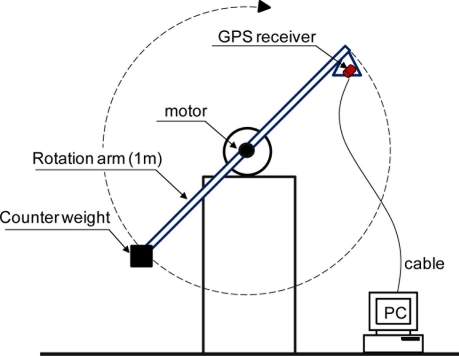
The dynamic simulator used in the laboratory experiments.

Laboratory experiments were designed to verify the ideas presented herein. A dynamic simulator shown in Figure 2 was developed to execute the experiments. This simulator enabled a uniform circular motion to be created in the vertical plane with specific periods. The arm of the simulator was 1.0 m, and the maximal vertical displacement was 2.0 m.

A high-resolution GPS receiver (NAVPAC VP-1000 model, ETEK Navigation, Inc.) was used in this study. The GPS receiver output latitude, longitude, time and velocity data via RS-232, and was therefore easily attachable to the data buoys. This GPS continued tracking satellites and relaying data even while the buoys were pitching and rolling violently under bad weather conditions. It has also proven capable of acquiring satellites and fixing on their positions very quickly, following a short period of signal obstruction. It is fully satisfied the requirements for the measurement of waves. The rotation periods tested in the experiments were 6, 7, 8, 9 and 10 s, which are within the frequency range of wind waves. GPS output velocities were acquired through a self-developed program. The sampling rate was 1 Hz, and twenty minutes of data was recorded for each test run.

**Figure 3.**
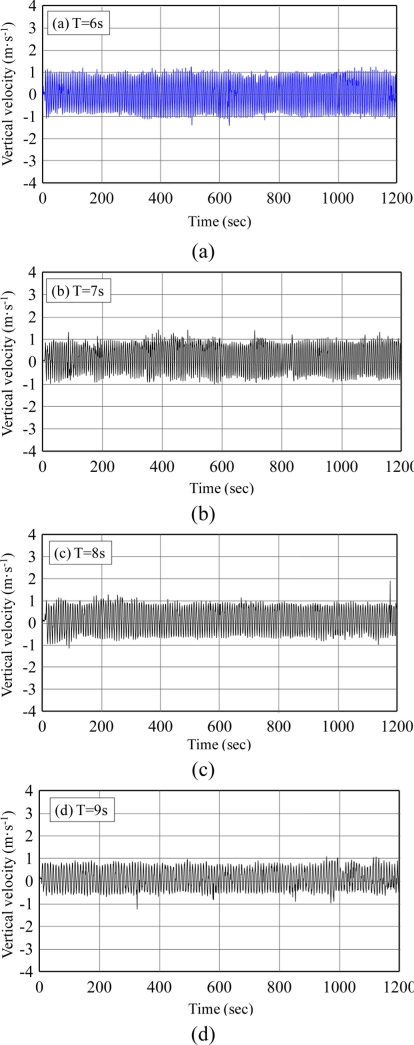
GPS vertical velocity output **(a)** T = 6 s; **(b)** T = 7 s; **(c)** T = 8 s; **(d)** T = 9 s.

**Figure 4.**
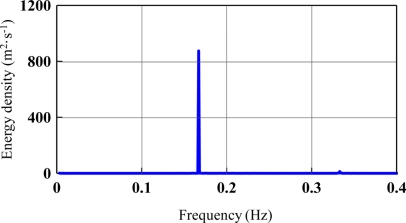
Vertical velocity spectrum (T = 6 s).

**Figure 5.**
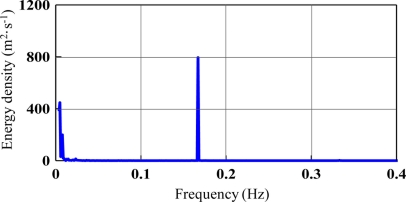
Vertical displacement spectrum (T = 6 s).

Figure 3(a–d) show the vertical velocities of all experimental cases. Harmonic motions were found. Fourier Transform was applied to obtain the velocity spectrum as shown in Figure 4, which is the case where rotation period equals 6 s. In Figure 4, we found the peak frequency locates at 0.167 Hz which equals the designed 6 s period. According to the transform function listed in Equation (6), the velocity spectrum was transferred to the displacement spectrum, as shown in Figure 5. The energy distribution in the very low frequency region was determined, with the exception of the anticipated energy distribution at a frequency of 0.167 Hz. This is the energy mapping of very high-speed satellite motion. Because the energy distribution of wave motion and satellite movement is located in significantly different frequency bands, it is very easy to separate them. For ocean waves, their energy distributes from 0.03 Hz to 0.4 Hz in the frequency band. In this study, the vertical displacement was derived by Equation (16):

(16) $$S\;(f_{0.03∼0.4\mathrm{Hz}})*\Delta f=\frac{1}{2}a^{2}$$

**Figure 6.**
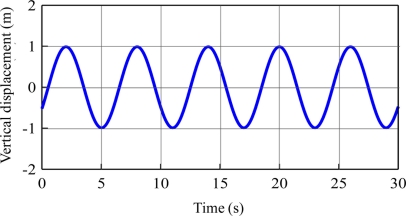
Inverse vertical displacement time series (T = 6 s).

The time series of vertical displacement is derived by the inverse transformation of the displacement spectrum. Figure 6 shows the inverse vertical displacement for the case in which T = 6 s. It has the same shape as the simulated one. The amplitude of the inverse vertical displacement was 1.97 m. The bias was 0.03 m (1.5%). The amplitude of the other 4 cases was 1.84 m, 1.90 m, 1.91 m, and 1.98 m, respectively. The average bias was 4% as shown in Table 1. These results show that this approach derived vertical displacement with a very high degree of precision and was able to eliminate the energy introduced by the motion of the satellite.

**Figure 7.**
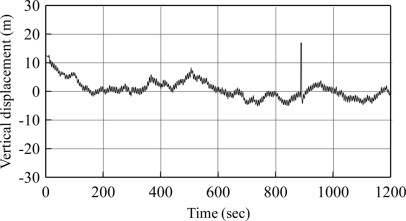
Vertical displacement outputted directly from GPS receiver (T = 6 s).

This paper presents an approach to derive vertical displacement from GPS velocity signals output. The vertical displacement data was used for the estimation of wave parameters. Two alternatives exist to obtain vertical displacement data. First, directly acquire the vertical displacement data from the GPS device. Second, integrate the vertical velocity to obtain displacement. Figure 7 shows the plot of direct vertical displacement from the GPS device. This was the output signal when executing the previous experiment in the case where T = 6 s. The condition of the experiment was such that vertical displacement was meant to vary within ±1.0 meter with regard to the length of the arm. In Figure 7, the direct output vertical displacement showed significant fluctuation, often exceeding the expected range. Without working with the base station, the vertical displacement of the GPS device showed a large degree of error with occasional spikes. This may have been caused by any number of factors. A large degree of error in the output of direct vertical displacement prohibits the measurement ocean wave motion with a GPS devise, using this approach.

**Figure 8.**
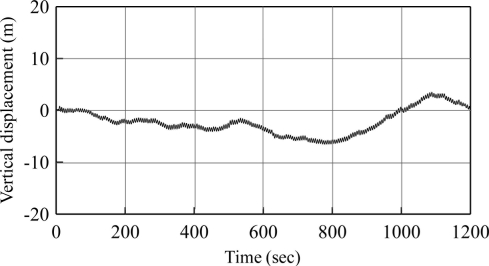
Vertical displacement derived from vertical velocity integration (T = 6 s).

Figure 8 shows the results of integrating the vertical velocity with displacement for the same case as previously used. The result presents a zero-drift caused by numerical integration. By applying the Fourier transform to this integrated time series, we discovered the peak frequency located at 0.17 Hz, which was the same as the input condition. Inverse Fourier transform was used to obtain the harmonic vibration again. The biases between simulated and derived amplitudes were 9.1%, 12.3%, 8.2%, 2.5%, and 3.0% for the five cases defined in this study. The average was 7.0%, higher than in the method presented in the previous section. This data shows again that our approach to obtaining vertical displacement through the spectral transformation from velocity to displacement is feasible for the measurement of ocean waves.

## 4. Field Tests

A GPS receiver was mounted on a data buoy deployed near Xiaoliuchiou Island in Southwestern Taiwan. The diameter of the accelerometer buoy was 2.5 m. It is one of the buoys in the network in Taiwanese waters. The water depth at the buoy location is 82 m. The buoy retained the original wave data and data from the meteorological sensors. The GPS output velocity signal was recorded by a logger on the buoy for comparison purposes. The field experiments were carried out from November 2006 to February 2007. A total of 2,023 simultaneous buoy and GPS measurements were collected.

### 4.1. The GPS Signal Drifting and Filter

**Figure 9.**
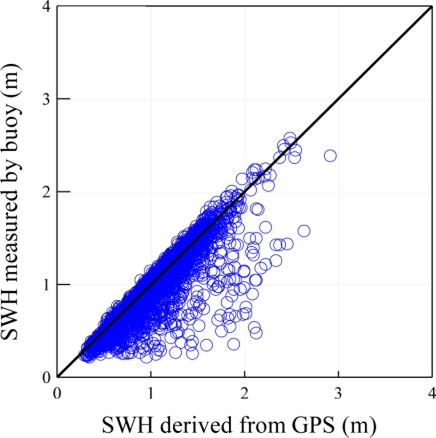
Comparison of significant wave heights from GPS derived and Buoy’s Accelerometer.

Comparative analysis showed that the correlation of simultaneous buoy data and GPS measurements was low. As shown in Figure 9 the coefficient of correlation was less than 0.5.

**Figure 10.**
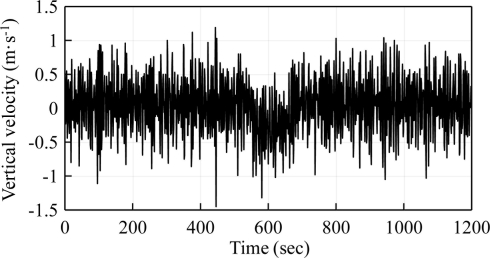
An example of the GPS output vertical velocity with signal drifting.

It was found that one of the causes was the occurrence of loss or drifting of the signal of GPS output velocity, as shown in Figure 10. This was due to the interruption of connections with GPS satellites or sheltering due to the motion of ocean waves. The moving average approach was applied to filter out this drifting data. Application of the moving average method with a window greater than five times the wave period showed significant improvements.

**Figure 11.**
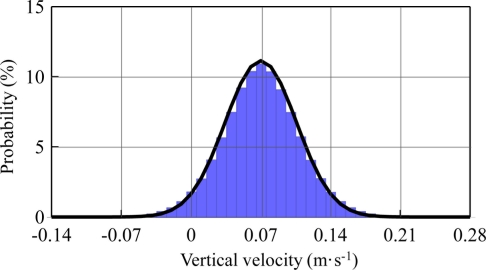
Probability distribution of the moving-averaged GPS output velocity (no signal loss or drifting).

Figure 11 shows the probability distribution of the moving-averaged GPS output velocity without signal drifting. This was verified as a normal distribution. Because the drifting data was unassociated with wave signals, they should not follow the normal distribution. GPS output velocities beyond three times the standard deviation from the moving average value have to be filtered. The confidence interval was therefore 99%. A total of 19 measurements were culled from within the 2,023 measurements. The data loss or drifting rate was less than 1.0%.

In addition, energy spikes occurring at very low frequencies caused by the transformation function were another cause for poor agreement between buoy and GPS measurements. The energy was amplified due to the −2 power of frequency in Equation (6) for some of the data. Noisy spectra derived erroneous wave parameters, and the frequency of gravity waves was generally greater than 0.03 Hz (30 s). This frequency was used as the criterion for frequency cut-off.

### 4.2. Validation of Wave Spectra and Parameters

A summary of the processes of wave measurement through GPS output velocity is shown below:

1. Acquisition of GPS output velocity.
2. Detecting and culling of data with signal loss or drifting.
3. Derivation of vertical spectrum.
4. Transformation from vertical spectrum to vertical displacement spectrum.
5. Derivation of wave parameters and one-dimensional spectrum.
6. Estimation of directional wave spectrum.

**Figure 12.**
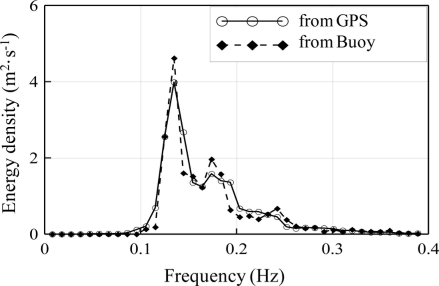
Comparison of the GPS-derived and buoy-measured frequency spectrum (Data: 2006/12/21 12:00, Hs = 1.4 m).

To study the performance of GPS wave measurements under various sea conditions, the comparative data was divided into four groups regarding *in-situ* significant wave height (SWH). They are the groups of SWH ≦ 1.0 m, 1.0 m < SWH ≦ 1.5 m, 1.5 m < SWH ≦ 2.0 m and SWH > 2.0 m. Figure 12 shows a comparison of the one-dimensional spectrum for the group of 1.0 m < SWH ≦ 1.5 m. We found very good agreement between the buoy and GPS measurements. The same results were found under other sea conditions.

**Figure 13.**
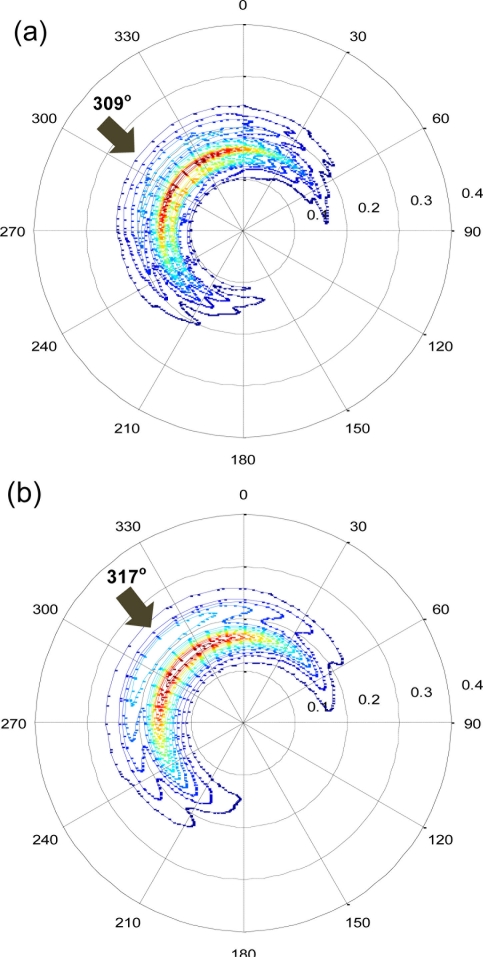
Comparison of the directional wave spectrum. **(a)** GPS-derived; **(b)** buoy-measured (Data: 2006/12/17 01:00).

Figure 13 shows the comparative results of the directional wave spectrum. The energy distribution was in general agreement, however, the mean bias of the wave direction was 10.5°. Although this result shows significant room for improvement, the degree (10.5°) was less than the angle of 32 cardinal directions that is satisfactory for a number of applications.

**Figure 14.**
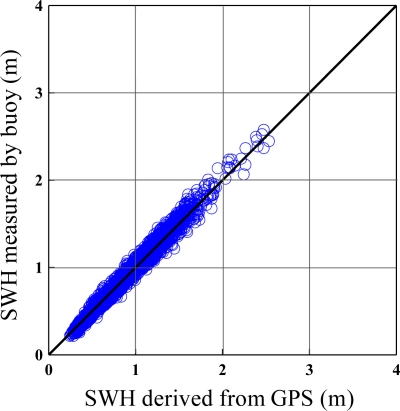
Comparison of significant wave height (SWH) between the buoy-measured with GPS-derived.

**Figure 15.**
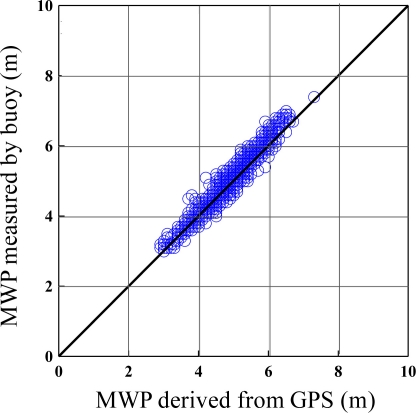
Comparison of mean wave period (MWP) between the buoy-measured with GPS-derived.

Verification of GPS-derived significant wave heights and mean wave periods are shown in Figures 14 and 15. Both Figures illustrate a correlation of over 0.95. The estimation of non-dimensional root mean square error is shown in Table 2, with 4.2% for the significant wave height and 4.4% for the mean period, respectively. It is clear that the wave parameters derived from the GPS were very much in agreement with the buoy measurements. According to sea conditions, it was found that the biases were between 4.5% and 3.6% with a significant wave height over 2 m. This means that the GPS-derived wave parameters had higher accuracy under severe sea conditions.

## 5. Conclusions

This study presented a methodology to derive wave parameters (e.g., significant wave heights, wave periods, direction, and wave spectrum) from GPS output velocities representing the velocity of water particles. The main concept behind this study was to transfer the velocity spectrum to the vertical displacement spectrum to obtain surface fluctuations. In addition, velocities in three-directions (heave, sway, and surge) were used to derive the directional wave spectrum. In addition to laboratory experiments, field tests were carried out to verify the accuracy of GPS-derived wave parameters. Results indicate that the vertical displacement obtained by the spectral transform was far more correct than either the direct GPS output or velocity integration. Signal loss and drifting as well as energy spikes at low frequencies induced by transformation were the cause of incorrect wave parameters. This study used the moving average skill and frequency cut-off to filter the data, and the filtered GPS-derived wave parameters showed good agreement with those measured on the buoy.

Comparisons of simultaneous GPS-derived and buoy-measured one-dimensional spectra and directional wave spectra showed good agreement. The coefficient of the correlation of wave parameters between these two devices was higher than 0.95. This verifies that the use of GPS output velocity to derive ocean wave parameters is a feasible option. Even though the mean bias of the wave direction comparison was still approximately 10°, it is still valuable for a number of purposes. We found that the biases between the GPS and buoy were quite stable, and did not change with changing sea conditions. This demonstrated that this method could be adopted for most wave conditions.

Results of this study have revealed that GPS has potential as an ocean wave measurement device in addition to its original positioning function, while conventional data buoys use only accelerometers to measure waves. Therefore, GPS receivers could be an alternative device for wave measurement thanks to their reasonable performance and low-cost.

## Figures and Tables

**Table 1.** Results of inversed vertical displacement experiments.

	Case 1	Case 2	Case 3	Case 4	Case 5
Conditions: Rotation period (sec) & frequency (Hz)	6 (0.17)	7 (0.14)	8 (0.13)	9 (0.11)	10 (0.1)
Peak frequency (Hz) of vertical displacement spectrum	0.17	0.14	0.13	0.11	0.10
Frequency bias	0%	0%	0%	0%	0%
Conditions: Twice of arm Length (m)	2.0	2.0	2.0	2.0	2.0
Amplitude (m) of inversed vertical displacement	1.97	1.84	1.90	1.91	1.98
Amplitude bias	1.5%	8.0%	5.0%	4.5%	1.0%

**Table 2.** Quantitative errors of GPS-derived wave parameters under various sea states.

Significant wave height	<1 m	1 ∼ 1.5 m	1.5 ∼ 2.0 m	>2.0 m	Average
bias	4.5%	4.0%	4.5%	3.6%	4.2%
Mean wave period	3 ∼ 4 s	4 ∼ 5 s	5 ∼ 6 s	>6 s	Average
bias	5.1%	4.1%	4.3%	4.0%	4.4%
